# Research on Mixing Uniformity Evaluation and Molding Method for Crumb Rubber Asphalt Mixtures

**DOI:** 10.3390/ma18225245

**Published:** 2025-11-20

**Authors:** Wenhua Wang, Yi Lu, Lingdi Kong, Wenke Yan, Yilong Li, Mulian Zheng, Chuan Lu, Guanglei Qu

**Affiliations:** 1 Shandong Expressway Infrastructure Construction Co., Ltd., Jinan 250000, China; 13774936743@163.com (W.W.); 15265910663@163.com (L.K.); y1065179163@163.com (Y.L.); 2 Shandong Expressway Shenhai Expressway Co., Ltd., Rizhao 276800, China; 3 Key Laboratory of Special Area Highway Engineering, Ministry of Education, Chang’an University, Xi’an 710064, China; 2023221251@chd.edu.cn (Y.L.); 2022221260@chd.edu.cn (W.Y.); quguanglei1216@163.com (G.Q.); 4 College of Civil Engineering, Qilu Institute of Technology, Jinan 250200, China; clu@chd.edu.cn; 5 School of Civil Engineering & Transportation, Beihua University, Jilin 132013, China

**Keywords:** road engineering, molding method, crumb rubber asphalt mixtures, orthogonal experiment, mixing uniformity

## Abstract

The broader adoption of crumb rubber asphalt mixtures (CRAM) as sustainable pavement materials is currently limited by two key technical barriers. Firstly, there is a lack of standardized methods to evaluate mixing uniformity. Secondly, the material’s tendency for elastic recovery after compaction remains problematic. These barriers ultimately hinder the realization of CRAM’s full potential in vibration reduction, noise abatement, and resource recycling. To improve the performance evaluation system of CRAM and promote its development in engineering applications. Based on the distribution characteristics of crumb rubber in asphalt mixtures, this study established a crumb rubber distribution area moment model. It proposed a coefficient of area–distance variation to evaluate the mixing uniformity of CRAM. Through compaction tests and orthogonal tests, the effects of mixing process, mixing time, mixing temperature, compaction temperature, compaction times, and compaction method on the mixing uniformity and performance of CRAM are systematically investigated. The results show that, compared with specimens prepared by single compaction and compaction after high-temperature curing, CRAM specimens prepared by secondary compaction exhibit superior mechanical performance. The 24 h elastic recovery rate of these specimens is reduced to 24% of that in single-compacted specimens. The mixing process and mixing time have a significant impact on the mixing uniformity of CRAM. Pre-mixing crumb rubber with aggregates or extending the mixing time can improve the CRAM mixing uniformity by 45% and 18%, respectively. The mixing and compaction temperatures primarily affect the bulk density and Marshall stability of the specimens. When the mixing and compaction temperatures are 180 °C and 170 °C, respectively, the bulk density and Marshall stability of the molded specimens reach their maximum values. Through orthogonal analysis, the optimal mixing method for CRAM is determined as follows: mix aggregates and crumb rubber at 180 °C for 40 s, then add asphalt and continue mixing for another 80 s. The optimal process for secondary compaction is as follows: the first compaction at 170 °C, compacting each side 47 times, and the second compaction at 80 °C, compacting each side 23 times.

## 1. Introduction

### 1.1. Background

With the rapid development of the social economy and the modern automobile industry, traffic volume and average vehicle speed continue to increase, resulting in more pronounced issues of traffic noise pollution and driving safety in winter. Traffic noise is not only a major source of environmental noise [1], but also poses a significant threat to the lives and health of residents along roadways. Statistics show that for every 1 decibel (dB) increase in regional noise levels, the risk of hypertension among local residents increases by approximately 3% [2,3,4]. In addition, approximately three-quarters of China’s territory experiences snow and ice during winter [5]. Compared with dry pavements, the skid resistance of snow-covered roads decreases by up to 65% [6]. Snow and ice accumulation on road surfaces has become the primary cause of frequent traffic accidents in winter [7,8,9]. In recent years, research on functional pavements has been continuously improved, and enhancing and expanding pavement functions has become a major focus in the international road engineering field [10]. From a pavement materials perspective, imparting high elasticity, high damping, and low modulus to pavements is an effective multi-functional approach. The high elasticity and low modulus contribute to de-icing in winter, while the high damping property reduces noise pollution during road operation [11].

Crumb Rubber Asphalt Mixtures (CRAM) are a new type of pavement material that incorporate crumb rubber into asphalt mixtures to partially replace fine aggregates [12,13,14,15]. CRAM pavements are characterized by low dynamic modulus, high damping, and strong elastic recovery ability [16,17]. During the interaction between CRAM and the tire, part of the tire’s vibrational energy is converted into heat and dissipated. This dissipation reduces the vibrations of both the vehicle body and the tire, shortens the duration of vibrations, and thereby achieves vibration reduction and noise mitigation [18,19]. During the winter, when roads are iced, the action of vehicle loads causes the elastic deformation of CRAM pavements, resulting in stress concentration in the ice layer. This alters the bond between the ice layer and the pavement, causing the ice layer to break. This enhances the road’s skid resistance and improves driving safety during winter [20,21,22].

However, the density of crumb rubber is only about half that of aggregates, making segregation prone to occur during the mixing of the mixtures [23]. Additionally, the high elasticity and high damping properties of crumb rubber can cause difficulties in compacting CRAM and elastic recovery after compaction [24], leading to increased void ratio in the specimens and reduced pavement performance. To address this issue, numerous researchers, both domestically and internationally, have conducted studies on the mixing and molding methods of CRAM, yielding specific results [25].

Bakheit et al. [26] proposed a complex mixing process for CRAM and compared it with dry and wet mixing processes. The results showed that the complex mixing process can improve the high-temperature stability and water stability of CRAM. Rodriguez-Fernandez et al. [27] found that CRAM prepared using the dry method exhibits lower aging levels and superior durability. Quan et al. [28] conducted orthogonal tests to investigate the effects of different mixing processes on the volume properties (density and air voids) of CRAM. The authors recommended heating the aggregates to 185 °C. The mixing sequence should be aggregates, crumb rubber, asphalt, and finally mineral powder. The crumb rubber should be mixed for 30 s, followed by the asphalt, with a total mixing time of 70 s. The entire process should be conducted at a mixing temperature of 170 °C.

In the molding process of CRAM, Farouk et al. [29] used gyratory compaction to mold CRAM specimens and found that the molded specimens achieved a target void ratio of 2% to 4%. Bueno et al. [30] found that placing CRAM in an oven for a short curing period before molding helps alleviate compaction difficulties. Yu et al. [31,32] optimized the molding process, with void ratio and expansion rate as the objectives. The optimal molding process for large Marshall specimens, their research determined, was secondary compaction: the first compaction occurred 20 times at a temperature of 140 °C, and the second compaction occurred 92 times at a temperature of 90 °C.

The above studies indicate that the mixing process has a significant impact on the high-temperature stability, water stability, durability, and volumetric properties (density and air voids) of CRAM. Dry, wet, and complex mixing processes each have their own characteristics, and pre-mixing rubber particles with aggregates helps improve the compactness of CRAM. Rotary compaction, vibratory compaction, secondary compaction, compaction after high-temperature curing, and increased compaction effort are all effective in optimizing the CRAM molding process. However, the use of rotary compaction and vibratory compaction equipment is less widespread. In contrast, secondary compaction and compaction after high-temperature curing methods can be performed using a Marshall compaction device, which is simpler to operate. These methods are more suitable for optimizing CRAM molding processes in a laboratory setting.

### 1.2. Objective and Scope

**Figure 1:**
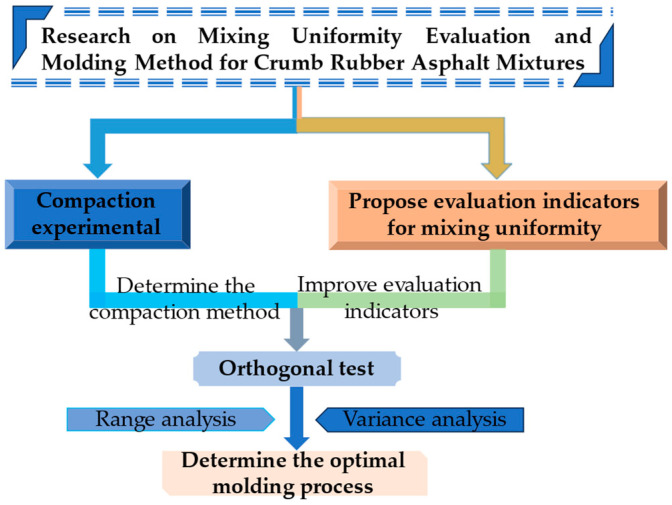
Research flowchart.

Currently, research on CRAM mixing methods remains incomplete, and effective evaluation methods for CRAM mixing uniformity are lacking. The molding process of CRAM has not yet been standardized, and studies by domestic and international scholars on the effects of secondary compaction temperature and compaction times on CRAM specimen performance are relatively limited. Therefore, this study proposes an evaluation method and model for ensuring the uniform mixing of CRAM. It further systematically investigates the CRAM molding method through compaction and orthogonal tests, followed by a comprehensive analysis of the factors affecting CRAM’s mixing and molding. Finally, based on the range and variance analyses of the orthogonal test data, an optimal molding method for CRAM is proposed. The research process is shown in Figure 1.

## 2. Materials and Gradation Design

### 2.1. Asphalt

The asphalt was SBSI-C modified asphalt produced by Qilu Petrochemical; its technical properties are shown in Table 1.

### 2.2. Aggregates

The aggregate was limestone produced by Shaanxi Xianyang Sand and Gravel Plant; its technical properties are shown in Table 2 and Table 3.

### 2.3. Crumb Rubber

**Figure 2:**
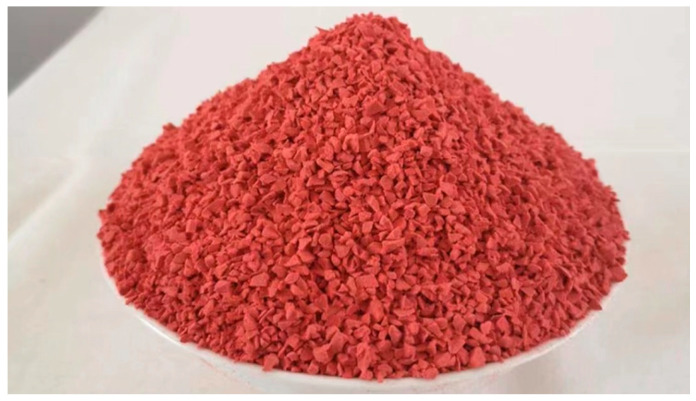
Bright orange EPDM crumb rubber.

This study selects bright orange ethylene propylene diene monomer (EPDM) crumb rubber, whose vivid color facilitates the subsequent identification and analysis of particle distribution uniformity in the asphalt mixtures, as shown in Figure 2. The crumb rubber size distribution and related technical properties of the EPDM crumb rubber are shown in Table 4 and Table 5.

### 2.4. Filler

The filler used was limestone powder produced by the Xianyang Sand and Gravel Plant, with the technical specifications shown in Table 6.

### 2.5. Crumb Rubber Asphalt Gradation Design

**Figure 3:**
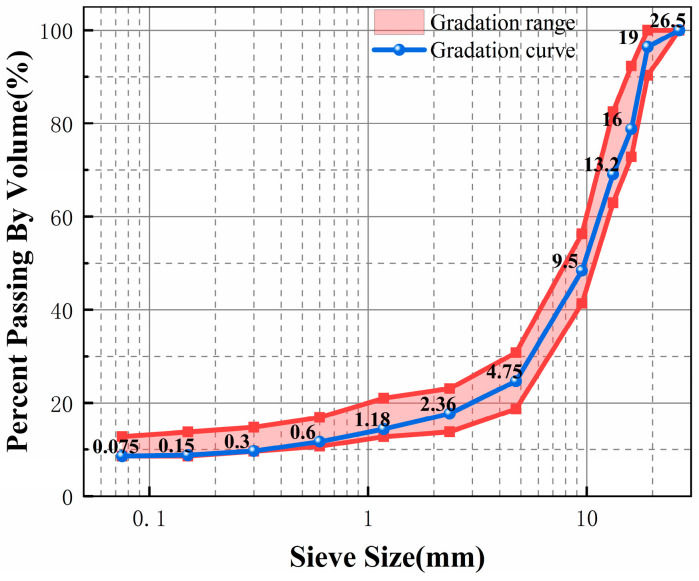
Crumb rubber asphalt mixtures’ gradation curve.

This study employed the SMA-20 gradation as recommended by the Technical Specifications for Construction of Highway Asphalt Pavements (JTGF40-2004) [33]. The crumb rubber content was set at 3% of the mass of the mineral aggregate. Based on these conditions, the CRAM gradation was calculated using the equal-volume replacement method. Table 7 and Figure 3 present the resulting gradation and gradation curve. Marshall tests determined the optimum asphalt–aggregate ratio for this gradation to be 4.3%. The gradation was calculated using the following method. Based on the specified crumb rubber content and the total mass of the original aggregate, the required mass of crumb rubber was determined. Then, the volume of the replaced aggregate was calculated based on its density, which represents the volume of crumb rubber to be added. Finally, the required mass of crumb rubber was calculated by converting the added volume using the crumb rubber density. The calculation process is shown in Equation (1).

(1) $$A=\frac{m_{r}}{m_{r}+M-m_{r}\times \frac{\rho_{s}}{\rho_{r}}}\times 100$$

where *A* is the crumb rubber content (mass ratio of crumb rubber to aggregates), %; *m_r_* is the mass of crumb rubber added, kg; *M* denotes the total mass of the original aggregates, kg; *ρ_s_* represents the density of the replaced aggregates, g/cm^3^; and *ρ_r_* refers to the density of the crumb rubber, g/cm^3^.

## 3. Experimental Design and Method

### 3.1. Evaluation Indicator for Crumb Rubber Mixing Uniformity

To address the lack of evaluation indicators for CRAM mixing uniformity, this study established a crumb rubber distribution area moment model based on the static moment theory. The concept and the calculation method of the coefficient of area–distance variation (C*_v_*) are introduced in this study. This utilizes relevant functions from the opencv library and digital image processing technology to quantitatively analyze the mixing uniformity of CRAM.

Evaluating the mixing uniformity of CRAM is crucial for revealing the effects of crumb rubber distribution on the mixture structure and pavement performance. It is also essential for ensuring the strength and durability of the mixtures and for preventing the early cracking and raveling caused by local segregation. Additionally, the uniformity indicator can provide quantitative guidance for optimizing the mixing process and controlling construction quality. This indicator represents a key technical aspect in the design and engineering application of CRAM. The C*_v_* is based on the area of crumb rubber and its distance to the boundary. It can directly capture the deviation of particles in local regions and reflect the spatial distribution characteristics of crumb rubber. It better represents the actual spatial variation in the material than a simple “number ratio” or “area ratio.” In addition, evaluating local non-uniformity through differences in the C*_v_* is essential for identifying “local crumb rubber enrichment zones” or “local crumb rubber deficiency zones.” After uploading the slice images, the coefficient can be automatically calculated in batches through a program. The operation is simple, facilitates experimental repetition, and is suitable for comparing different mix designs and mixing processes.

**Figure 4:**
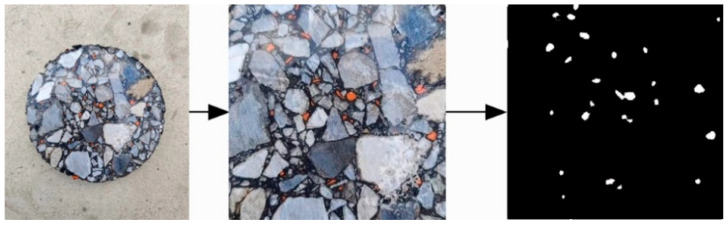
Schematic diagram of image preprocessing.

The image preprocessing technique used is shown in Figure 4. First, the image of the cross-section at half the height of the specimen is captured, with the center of the inner circle serving as the midpoint. An inscribed square is then extracted from this cross-section image. Next, the extracted image is resized to 450 pixels (px) × 450 pixels (px). Finally, the cropped image undergoes binarization, where the crumb rubber is colored white, and the remaining areas are colored black.

**Figure 5:**
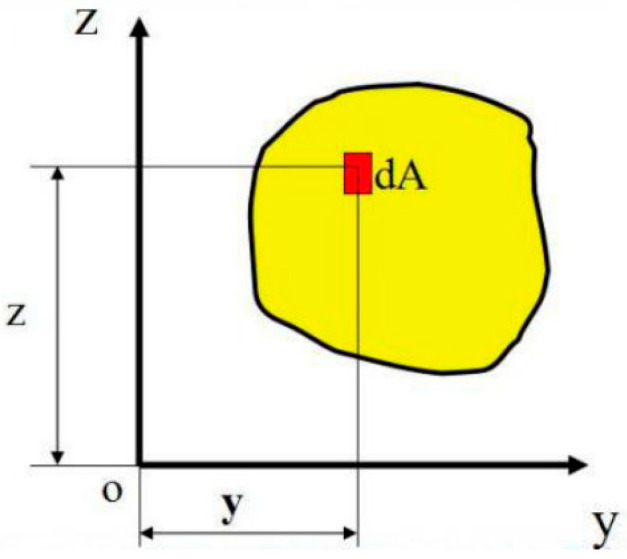
Schematic diagram of area moments.

Based on the distribution characteristics of crumb rubber in the binarized image, a crumb rubber distribution area moment model is established. The area moment (S) is defined as the product of the particle area (A) and the distance (d) from the particle’s centroid to the corresponding coordinate axis. The schematic diagram and calculation method are shown in Figure 5 and Equations (2) and (3).

(2) $$S_{Z}=\int_{A}ydA$$

(3) $$S_{y}=\int_{A}zdA$$

**Figure 6:**
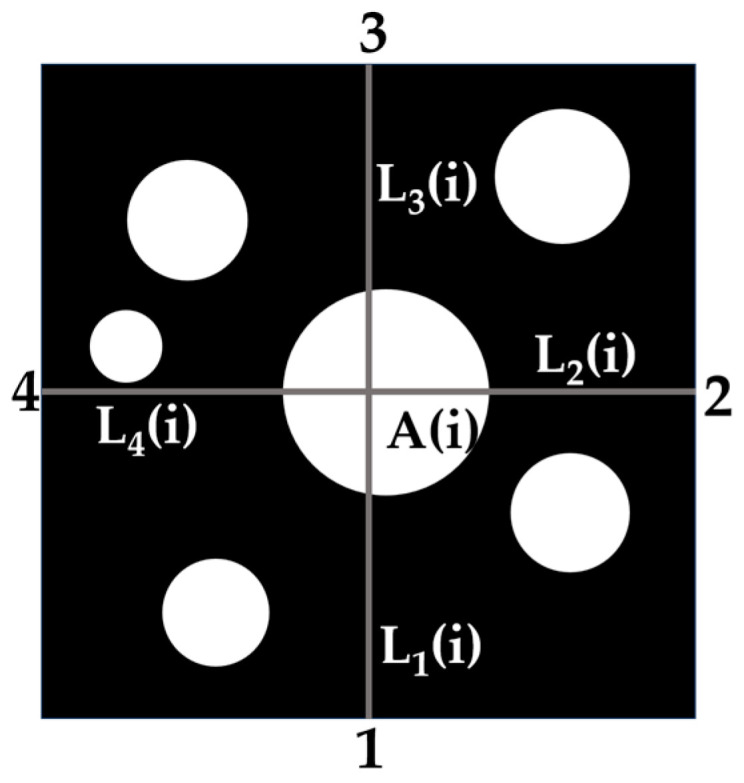
Schematic diagram of four-sided area moments.

The quadrilateral area moment for crumb rubber in the Marshall specimen cross-section refers to the sum of area–distance between all particles and a specified image boundary. Figure 6 and Equation (4) illustrate the schematic and calculation method.

(4) $$S_{x}=\sum_{i=1}^{n}\left(s(i)\times l_{x}(i)\right)$$

where *S_x_* denotes the total area moment of crumb rubber relative to image boundary *x* (*x* = 1, 2, 3, 4), in units of px^3^; *s*(*i*) represents the area of the *i*-th crumb rubber in the cross-section, in units of px^2^; *l_x_*(*i*) refers to the distance from the centroid of the *i*-th crumb rubber to the *x*-th boundary, in pixels (px); *n* indicates the amount of crumb rubber within the cross-section.

Based on the crumb rubber area moment model, this study proposes using the coefficient of area–distance variation (*C_v_*) to evaluate the uniformity of crumb rubber distribution within the mixtures. Equations (5) and (6) show the calculation process for *C_v_* and Sb. A higher *C_v_* reflects more severe segregation during CRAM mixing; a lower *C_v_* indicates better uniformity.

(5) $$C_{v}=\frac{\sqrt{\frac{{\left(S_{1}-S_{b}\right)}^{2}+{\left(S_{2}-S_{b}\right)}^{2}+{\left(S_{3}-S_{b}\right)}^{2}+{\left(S_{4}-S_{b}\right)}^{2}}{4}}}{S_{b}}$$

(6) $$S_{b}=\frac{n^{2}}{2}sl$$

where *S*_1–4_ denotes the area–distance sum of crumb rubber relative to boundaries 1 to 4, respectively, in units of px^3^; *S_b_* refers to the area–distance sum of crumb rubber under uniform distribution relative to any boundary, in units of px^3^; *n* represents the number of crumb rubber per row (or column) in the uniform distribution; *s* indicates the area of crumb rubber under uniform distribution, in units of px^2^; *l* refers to the side length of the square image being cut, in units of pixels (px).

### 3.2. Crumb Rubber Asphalt Mixtures Molding Experimental Design

#### 3.2.1. Compaction Experimental Design

To address the elastic recovery of CRAM specimens after compaction, this study uses three forming methods: single compaction, secondary compaction, and compaction after high-temperature curing to prepare Marshall specimens. By comparing the changes in the void ratio, Marshall stability, and 24 h elastic recovery rate of specimens under different compaction methods, the optimal compaction method for CRAM is determined.

**Figure 7:**
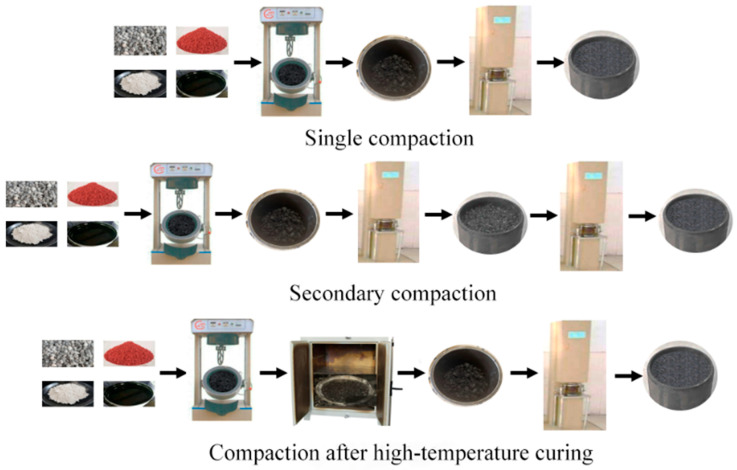
Description of molding methods.

The mixing procedure followed the BH-3 test scheme outlined in Section 3.2.2. First, aggregates and crumb rubber were pre-mixed at 180 °C for 40 s. Then, asphalt was added and mixed for 80 s, followed by the addition of mineral powder for a final 60 s of mixing. The compaction steps and parameter settings are shown in Figure 7 and Table 8. For high-temperature curing followed by the compaction method, the curing temperature and time were set at 170 °C and 30 min, respectively. The calculation method for the 24 h elastic recovery rate is given in Equation (7).

(7) $$R_{24h}=\frac{\left(h_{2}-h_{1}\right)}{h_{1}}$$

where *R*_24h_ represents the 24 h elastic recovery rate of Marshall specimens, in percentage (%); *h*_1_ refers to the height of the Marshall specimen after molding, in millimeters (mm); *h*_2_ denotes the height of the Marshall specimen 24 h after compaction, in millimeters (mm).

#### 3.2.2. Orthogonal Test Design

**Figure 8:**
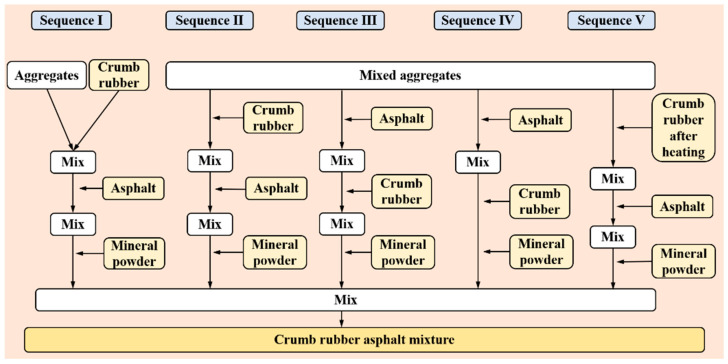
Mixing process flowchart.

To investigate the influence of mixing methods on the performance of CRAM Marshall specimens and the mixing uniformity, an orthogonal experiment was designed [41,42]. The experiment considered five key factors: mixing process, mixing time after adding crumb rubber, mixing time after adding asphalt, mixing temperature, and compaction temperature. Each factor was set at five representative levels, as shown in Table 9. The mixing process is illustrated in Figure 8. The orthogonal experimental scheme is shown in Table 10. CRAM Marshall specimen performance and crumb rubber distribution uniformity were evaluated as bulk density, void ratio, Marshall stability, and coefficient of area–distance variation. Tests for void ratio, bulk density, and Marshall stability follow methods T0707, T0708, and T0709 in Test Methods for Bitumen and Bituminous Mixtures in Highway Engineering (JTG E20-2011) [43].

All specimens in the orthogonal test were formed using the secondary compaction method. The first-stage compaction followed the compaction temperature listed in Table 10, while the second-stage compaction was carried out at 80 °C. The numbers of blows for the first and second stages are 47 and 23, respectively.

## 4. Experimental Results and Analysis

### 4.1. Analysis of Compaction Experiment Results

**Figure 9:**
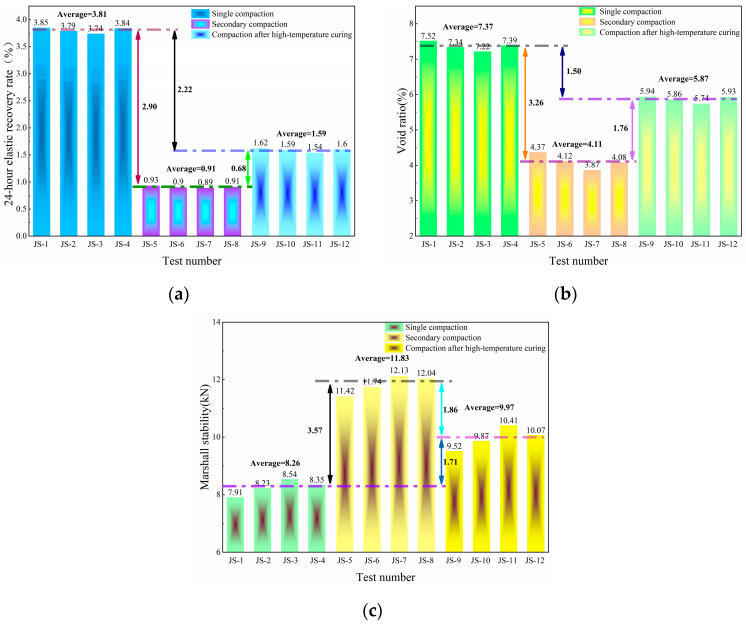
Compaction test results (**a**) 24 h elastic recovery rate; (**b**) Void ratio; (**c**) Marshall stability.

Based on the compaction parameters listed in Table 8, the study prepares the corresponding Marshall specimens and measures their void ratio, Marshall stability, and 24 h elastic recovery rate. The test results are presented in Figure 9.

Compared to single compaction, both secondary compaction and compaction after high-temperature curing effectively mitigate elastic recovery. Among these, the secondary compaction method yields the best results, reducing the specimen’s 24 h elastic recovery rate by 76%.

The elastic recovery of CRAM mainly results from the high internal temperature of the specimen after compaction, which causes the compressed crumb rubber to expand. Meanwhile, the reduced viscosity of the asphalt leads to insufficient restraint, making it difficult to restrain the deformation of the crumb rubber. The specimen experienced elastic recovery, which led to reduced compactness and increased height. The secondary compaction method helps eliminate the elastic recovery that occurs as the specimen cools to the second compaction temperature. In addition, the lower internal temperature after the second compaction reduces the risk of further rebound.

However, when the total number of compaction times is less than 70, the 24 h elastic recovery rate and void ratio of the specimen gradually decrease with increasing compaction times, while the Marshall stability gradually increases. At 70 compaction times, the specimen prepared by secondary compaction achieves the lowest 24 h elastic recovery rate and void ratio (0.89% and 3.87%, respectively), as well as the highest Marshall stability (12.13 kN). When the number of compaction times reaches 75, the surface aggregates of the specimen are crushed, leading to a decline in all performance indicators. Therefore, this study recommends using the secondary compaction method for CRAM specimen preparation, with 47 times for the first compaction and 23 times for the second compaction.

### 4.2. Analysis of Orthogonal Test Results

#### 4.2.1. Range Analysis

**Figure 10:**
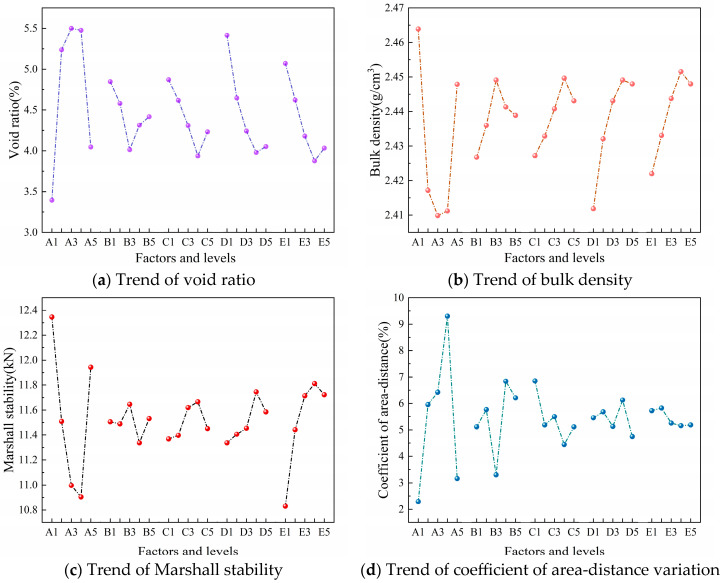
Trend map of evaluation indicators.

To determine the priority of each factor’s influence on the evaluation indicators, this study conducted a range analysis of the orthogonal test results. The orthogonal test results and range analysis are presented in Table 11 and Table 12, and the trends of the evaluation indicators are shown in Figure 10.

Changes in the mixing process directly affect the uniformity of crumb rubber distribution within the mixtures. The uneven distribution of crumb rubber is the main reason for the increased void ratio in CRAM specimens, as well as the reduced compactness and overall strength. In the CRAM mixing process, if crumb rubber is added after the asphalt, the high viscosity of the asphalt tends to cause crumb agglomeration [44,45], which hinders their uniform dispersion within the mixtures. If the aggregates are mixed first before adding crumb rubber, the mixing time is prolonged, which affects production efficiency. Moreover, since the distribution of aggregates becomes relatively fixed after mixing, and crumb rubber has a lower density and mass, the crumb rubber particles tend to accumulate in the upper part of the mixtures after mixing [46,47].

Mixing temperature has a significant impact on the void ratio and bulk density of CRAM specimens. As the temperature increases, asphalt viscosity decreases, and fluidity improves, which facilitates the more effective filling of voids between aggregates, thereby reducing the void ratio and increasing specimen compactness. Additionally, increasing the mixing temperature appropriately helps asphalt coat the aggregates more effectively, thereby enhancing the cohesion of CRAM [48]. However, an excessively high mixing temperature accelerates asphalt aging and causes excessive carbonization of crumb rubber, which adversely affects the bonding performance of the mixtures. Compaction temperature has a significant effect on the Marshall stability of CRAM specimens. If the compaction temperature is too low, the viscosity of the asphalt increases, making the mixtures difficult to compact. When the compaction temperature is too high, crumb rubber exhibits strong elastic recovery, and the low asphalt viscosity provides insufficient confinement [49]. This combination ultimately leads to elastic recovery in the specimen after compaction. Therefore, it is necessary to select appropriate mixing and compaction temperatures during the CRAM mixing process to enhance the strength and compactness of the specimens.

Mixing time has a significant impact on the distribution uniformity of crumb rubber in CRAM. If the mixing time is too short, crumb rubber, aggregates, and asphalt cannot blend thoroughly, increasing the coefficient of area–distance variation in the CRAM specimen and potentially causing poorly coated mixtures. An excessive mixing time leads to the over-carbonization of crumb rubber, increasing its hardness and adversely affecting the bonding performance of the mixtures [50].

According to the range analysis results, the factor importance ranking influencing CRAM mixing and compaction is mixing process > compaction temperature > mixing temperature > rubber particle mixing time > asphalt mixing time (AEDBC). The priority ranking of factor effects on evaluation indicators is shown in Table 13.

This study evaluated the performance of CRAM using void ratio, bulk density, Marshall stability, and coefficient of area–distance variation. At present, there is an absence of standardized specifications defining the appropriate ranges for the void ratio and Marshall stability of CRAM. The gradation of CRAM was derived by adjusting the SMA-20 gradation, and SBS asphalt was used. Therefore, the relevant parameters of CRAM Marshall specimens should meet the technical requirements for SMA Marshall mixtures’ design. Based on the principles of low void ratio, high density, high Marshall stability, and low coefficient of area–distance variation, the optimal factor-level combinations for each evaluation indicator were determined, as shown in Table 14.

#### 4.2.2. Variance Analysis

To determine whether each factor has a significant effect on the evaluation indicators and to enhance the reliability of the optimization results, this study conducted an analysis of variance on the orthogonal test data. The results are presented in Table 15, Table 16, Table 17 and Table 18. The mixing process has a highly significant effect on the Marshall stability and coefficient of the area–distance variation in CRAM specimens, and a significant effect on bulk density and void ratio. The mixing times for adding crumb rubber and asphalt have highly significant and significant effects, respectively, on the coefficient of area–distance variation in CRAM specimens, while their effects on other evaluation indicators are not significant. Mixing temperature and compaction temperature have a relatively significant and significant effect, respectively, on the bulk density and Marshall stability of CRAM specimens, while their effects on other evaluation indicators are not significant.

#### 4.2.3. Comprehensive Analysis

Range and variance analyses of the orthogonal test results indicate that the mixing process is the most significant factor affecting void ratio, bulk density, Marshall stability, and the coefficient of area–distance variation. The absence of asphalt and mineral powder during the pre-mixing of crumb rubber and aggregates reduces mixing resistance, allowing crumb rubber to disperse more fully. Improved CRAM mixing uniformity results in a decreased void ratio and decreased coefficient of area–distance variation, as well as increased Marshall stability and bulk density. Therefore, the optimal mixing process for CRAM is to first add crumb rubber and aggregates into the mixer for pre-mixing. Then, asphalt can be added for continued mixing, followed finally by the mineral powder to achieve uniform mixing.

The mixing time after adding crumb rubber and the mixing time after adding asphalt have significant effects on the CRAM mixing uniformity, while their effects on other evaluation indicators are not significant. Moderately extending the mixing time can improve the mixing uniformity of CRAM; however, excessive mixing time reduces mixing efficiency and increases energy consumption during the process. When the crumb rubber mixing time is at the B3 level and the asphalt mixing time is at the C4 level, the coefficient of area–distance variation and void ratio of the molded specimens reach their minimum values. Therefore, it is determined that the crumb rubber mixing time and the asphalt mixing time during CRAM mixing should be 40 s and 80 s, respectively.

Mixing temperature has a relatively significant effect on the bulk density of CRAM specimens, while compaction temperature has a significant effect on their Marshall stability. When the mixing temperature is set at level D4 and the compaction temperature at level E4, the asphalt adheres more effectively to the surfaces of aggregates and crumb rubber. This results in molded specimens that achieve the maximum bulk density and Marshall stability. In addition, excessively high mixing and compaction temperatures can accelerate the carbonization of crumb rubber and the aging of asphalt, thereby impairing the vibration-damping, noise-reduction, and mechanical performance of CRAM. Therefore, the optimal mixing temperature for CRAM is determined to be 180 °C, with the optimal first and second compaction temperatures set at 170 °C and 80 °C, respectively.

Based on the results of the range and variance analyses from the orthogonal tests, the optimal mixing and compaction method for CRAM was determined. This method involves mixing aggregates and crumb rubber at 180 °C for 40 s, then adding asphalt and continuing to mix for another 80 s. The specimen molding uses a secondary compaction method, applying the first compaction at 170 °C and the second at 80 °C.

## 5. Conclusions

To address the lack of evaluation indicators for CRAM mixing uniformity and the problem of elastic recovery after specimen compaction, this study first introduces the coefficient of area–distance variation to evaluate CRAM mixing uniformity. Subsequently, compaction tests and orthogonal experiments are conducted to investigate the molding method of CRAM Marshall specimens. The results indicate that the following:

(1) To quantitatively analyze CRAM mixing uniformity, this study establishes a crumb rubber distribution area moment model based on static moment theory and digital image processing technology. Additionally, it proposes the coefficient of area–distance variation (*C_v_*) along with the corresponding calculation method.

(2) The secondary compaction method significantly reduces elastic recovery and void ratio while enhancing Marshall stability. Based on these comprehensive improvements, it is therefore recommended as the molding method for CRAM specimens.

(3) Pre-mixing crumb rubber with aggregates and appropriately extending the mixing time can optimize the void ratio, bulk density, Marshall stability, and mixing uniformity of CRAM. The mixing and compaction temperatures mainly affect the density and Marshall stability of CRAM.

(4) The optimal molding method for CRAM involves mixing aggregates with crumb rubber at 180 °C for 40 s, followed by the addition of asphalt and continued mixing for an additional 80 s. This should be applied 47 times to both sides at 170 °C in the first stage and 23 times to both sides at 80 °C in the second stage.

(5) This study is of significant importance for enhancing the design standards, construction quality, and road performance of CRAM. Subsequent research can build upon the findings presented herein to propose quality control standards for CRAM, thereby promoting their standardized and normative application.

## Figures and Tables

**Table 1:** Test results showing SBSI-C asphalt’s technical properties.

Test Properties	Requirements	Test Results	Test Method
Penetration (25 °C, 0.1 mm)	60~80	68	JTG F40-T0604 [33]
Softening point (°C)	>55	84	JTG F40-T0606
Ductility (5 °C, cm)	>30	40.7	JTG F40-T0605
Flash point (°C)	>230	292	JTG F40-T0611
Density (15 °C, g·cm^−3^)	Measured	0.925	JTG F40-T0603
After RTFOT			
Mass change (%)	±1.0	−0.208	JTG F40-T0610
Penetration ratio (%)	>61	66	JTG F40-T0604
Ductility (5 °C, cm)	>20	31	JTG F40-T0605

**Table 2:** Test results showing technical properties of coarse aggregates.

Test Properties		Requirements	Test Results	Test Method
Aggregate crushing value (%)		≤26	12.3	JTG 3432-T0316 [34]
Los Angeles abrasion loss (%)		≤28	11.8	JTG 3432-T0317
Soundness (%)		≤12	7.1	JTG 3432-T0314
Flat and elongated particle content (%)		≤15	7.4	JTG 3432-T0312
Adhesion with asphalt		≥Level 5	Level 5	JTG 3432-T0616
Water washing method ≮ 0.075 Particle content (%)		≤0.8	0.3	JTG 3432-T0310
Apparent specific gravity (g·cm^−3^)	19~26.5 mm	≥2.6	2.82	JTG 3432-T0304
	16~19 mm		2.78	
	13.2~16 mm		2.79	
	9.5~13.2 mm		2.66	
	4.75~9.5 mm		2.80	

**Table 3:** Test results showing technical properties of fine aggregates.

Test Properties	Requirements	Test Results	Test Method
Apparent specific gravity (g·cm^−3^)	≥2.5	2.63	JTG 3432-T0328
Soundness (%)	≥12	14.2	JTG 3432-T0340
Mud content (%)	≤15	8.4	JTG 3432-T0333
Sand equivalent value (%)	≥60	74	JTG 3432-T0334

**Table 4:** EPDM crumb rubber size distribution.

Sieve Size (mm)	Percent Passing (%)	Test Method
>4.75	0	JTG 3432-T0307
2.36~4.75	66.9	
1.18~2.36	30	

**Table 5:** Technical properties of EPDM crumb rubber.

Test Properties	Test Results	Test Method
Water content (%)	0.16	GB/T 19208-6.1.1 [35]
Apparent specific gravity (g·cm^−3^)	1.43	GB/T 6343 [36]
Fiber and impurity content (%)	0.34	JT/T 797-6.2.3 [37]
Rubber hydrocarbon content (%)	22	ASTM D297 [38]
Flat and elongated particles content (%)	15	ASTM D4791 [39]
Shore A hardness (%)	67	ASTM D2240 [40]

**Table 6:** Test results showing the technical properties of filler.

Test Properties	Requirements	Test Results	Test Method
Apparent specific gravity (g·cm^−3^)	>2.5	2.54	JTG 3432-T0352
Water content (%)	<1	0.6	JTG 3432-T0103
Appearance	No granular agglomeration observed	No granular agglomeration observed	-
Hydrophilic coefficient	<1	0.5	JTG 3432-T0353

**Table 7:** Crumb rubber asphalt mixtures gradation.

Sieve Size (mm)	26.5	19	16.0	13.2	9.5	4.75	2.36	1.18	0.6	0.3	0.15	0.075
Percent passing by volume (%)	100	96.4	78.8	69.1	48.4	24.7	17.7	14.4	11.7	9.7	8.8	8.6

**Table 8:** Selected molding process parameters.

Test Number	Compaction Method	Compaction Temperature		Compaction Times	
		The First-Stage Compaction (°C)	The Second-Stage Compaction (°C)	The First-Stage Compaction (Time)	The Second-Stage Compaction (Time)
JS-1	Single compaction	170	n/a	60	n/a
JS-2				65	
JS-3				70	
JS-4				75	
JS-5	Secondary compaction	170	80	40	20
JS-6				43	22
JS-7				47	23
JS-8				50	25
JS-9	Compaction after high-temperature curing	170	n/a	60	n/a
JS-10				65	
JS-11				70	
JS-12				75	

**Table 9:** Factors and levels of orthogonal design.

Factors	Symbol	Levels				
		1	2	3	4	5
Mixing process	A	Sequence Ⅰ	Sequence Ⅱ	Sequence Ⅲ	Sequence Ⅳ	Sequence Ⅴ
Mixing time after adding crumb rubber (s)	B	20	30	40	50	60
Mixing time after adding asphalt (s)	C	50	60	70	80	90
Mixing temperature (°C)	D	150	160	170	180	190
Compaction temperature (°C)	E	140	150	160	170	180

**Table 10:** Scheme of orthogonal test.

Test Number	Mixing Process	Mixing Time After Adding Crumb Rubber (s)	Mixing Temperature (°C)	Compaction Temperature (°C)	Mixing Time After Adding Asphalt (s)
BH-1	Ⅰ	20	150	140	50
BH-2	Ⅰ	30	170	170	60
BH-3	Ⅰ	40	190	150	70
BH-4	Ⅰ	50	160	180	80
BH-5	Ⅰ	60	180	160	90
BH-6	Ⅱ	20	190	170	90
BH-7	Ⅱ	30	160	150	50
BH-8	Ⅱ	40	180	180	60
BH-9	Ⅱ	50	150	160	70
BH-10	Ⅱ	60	170	140	80
BH-11	Ⅲ	20	180	150	80
BH-12	Ⅲ	30	150	180	90
BH-13	Ⅲ	40	170	160	50
BH-14	Ⅲ	50	190	140	60
BH-15	Ⅲ	60	160	170	70
BH-16	Ⅳ	20	170	180	70
BH-17	Ⅳ	30	190	160	80
BH-18	Ⅳ	40	160	140	90
BH-19	Ⅳ	50	180	170	50
BH-20	Ⅳ	60	150	150	60
BH-21	Ⅴ	20	160	160	60
BH-22	Ⅴ	30	180	140	70
BH-23	Ⅴ	40	150	170	80
BH-24	Ⅴ	50	170	150	90
BH-25	Ⅴ	60	190	180	50

**Table 11:** Orthogonal test results.

Test Number	Void Ratio (%)	Bulk Density (g·cm^−3^)	Marshall Stability (kN)	Coefficient of Area–Distance Variation (%)
BH-1	3.40	2.460	12.350	23.49
BH-2	4.84	2.425	11.508	31.33
BH-3	4.87	2.427	11.368	36.22
BH-4	5.41	2.412	11.334	32.29
BH-5	5.07	2.422	10.826	33.15
BH-6	5.24	2.417	11.508	33.81
BH-7	4.58	2.436	11.494	33.16
BH-8	4.62	2.433	11.398	31.72
BH-9	4.65	2.432	11.404	32.97
BH-10	4.63	2.433	11.448	33.36
BH-11	5.50	2.410	11.000	35.05
BH-12	4.01	2.449	11.652	26.31
BH-13	4.32	2.441	11.618	32.52
BH-14	4.24	2.443	11.456	31.40
BH-15	4.18	2.444	11.710	31.79
BH-16	5.48	2.411	10.902	43.06
BH-17	4.32	2.441	11.334	36.13
BH-18	3.95	2.450	11.668	29.56
BH-19	3.98	2.449	11.744	34.27
BH-20	3.88	2.452	11.816	31.51
BH-21	4.04	2.448	11.942	25.93
BH-22	4.41	2.439	11.532	34.40
BH-23	4.23	2.443	11.448	31.32
BH-24	4.05	2.448	11.582	30.37
BH-25	4.04	2.448	11.720	31.53

**Table 12:** Range analysis of test results.

Factors	Range(R)			
	Void Ratio	Bulk Density	Marshall Stability	Coefficient of Area–Distance Variation
Mixing process	2.11	0.054	1.448	17.13
Mixing time after adding crumb rubber	0.83	0.024	0.318	9.82
Mixing time after adding asphalt	0.92	0.023	0.32	2.95
Mixing temperature	1.43	0.037	0.41	3.92
Compaction temperature	1.19	0.03	0.99	1.85

**Table 13:** Priority order of the impact of factors on evaluation indicators.

Evaluation Indicators	Priority Order of Impact of Factors
Void ratio	A > D > E > C > B
Bulk density	A > D > E > B > C
Marshall stability	A > E > D > B > C
Coefficient of area–distance variation	A > B > C > D > E

**Table 14:** Optimal combination of factor levels.

Evaluation Indicators	Optimal Combination
Void ratio	A1B3C4D4E4
Bulk density	A1B3C4D4E4
Marshall stability	A1B3C4D4E4
Coefficient of area–distance variation	A1B3C4D5E4

**Table 15:** Variance analysis of void ratio.

Factors	Sum of Squared Deviations	df	F	*p*	Significant
A	18.302	4	9.65	0.025	**
B	1.897	4	1.00	0.500	
C	2.535	4	1.34	0.393	
D	6.927	4	3.65	0.119	
E	4.711	4	2.48	0.200	
e	1.897	4			

**: relatively significant.

**Table 16:** Variance analysis of Gross bulk density.

Factors	Sum of Squared Deviations	df	F	*p*	Significant
A	0.01205	4	9.444	0.0257	**
B	0.00128	4	1.000	0.5000	
C	0.00160	4	1.257	0.4150	
D	0.00475	4	4.187	0.0972	*
E	0.00298	4	2.339	0.2154	
e	0.00128	4			

*: significant; **: relatively significant.

**Table 17:** Variance analysis of Marshallian stability.

Factors	Sum of Squared Deviations	df	F	*p*	Significant
A	1.517	4	22.465	0.0053	***
B	0.068	4	1.000	0.5000	
C	0.073	4	1.076	0.4725	
D	0.105	4	1.553	0.3401	
E	0.649	4	9.612	0.0250	**
e	0.068	4			

**: relatively significant; ***: most significant.

**Table 18:** Variance analysis of coefficient of area–distance variation.

Factors	Sum of Squared Deviations	df	F	*p*	Significant
A	243.796	4	73.479	0.001	***
B	56.60	4	17.068	0.009	***
C	24.16	4	7.298	0.040	**
D	8.94	4	2.669	0.182	
E	3.11	4	1.000	0.500	
e	3.11	4			

**: relatively significant; ***: most significant.

## Data Availability

The original contributions presented in this study are included in the article. Further inquiries can be directed to the corresponding author.

## Abbreviations

The following abbreviations are used in this manuscript:

CRAM	Crumb Rubber Asphalt Mixtures
dB	Decibel
EPDM	Ethylene Propylene Diene Monomer
SMA	Stone Mastic Asphalt
px	Pixel
